# Flexible Enantioselectivity of Tryptophanase Attributable to Benzene Ring in Heterocyclic Moiety of D-Tryptophan 

**DOI:** 10.3390/life2020215

**Published:** 2012-05-30

**Authors:** Akihiko Shimada, Haruka Ozaki

**Affiliations:** Sustainable Environmental Studies, Graduate School of Life and Environmental Sciences, University of Tsukuba, Tsukuba, Ibaraki 305-8572, Japan; E-Mail: ozaki.haruka@wako-chem.co.jp

**Keywords:** tryptophanase, D-tryptophan, flexible stereoselectivity, diammoniumhydrogen phosphate, origin of homochirality

## Abstract

The invariance principle of enzyme enantioselectivity must be absolute because it is absolutely essential to the homochiral biological world. Most enzymes are strictly enantioselective, and tryptophanase is one of the enzymes with extreme absolute enantioselectivity for L-tryptophan. Contrary to conventional knowledge about the principle, tryptophanase becomes flexible to catalyze D-tryptophan in the presence of diammonium hydrogenphosphate. Since D-amino acids are ordinarily inert or function as inhibitors even though they are bound to the active site, the inhibition behavior of D-tryptophan and several inhibitors involved in this process was examined in terms of kinetics to explain the reason for this flexible enantioselectivity in the presence of diammonium hydrogenphosphate. Diammonium hydrogenphosphate gave tryptophanase a small conformational change so that D-tryptophan could work as a substrate. As opposed to other D-amino acids, D-tryptophan is a very bulky amino acid with a benzene ring in its heterocyclic moiety, and so we suggest that this structural feature makes the catalysis of D-tryptophan degradation possible, consequently leading to the flexible enantioselectivity. The present results not only help to understand the mechanism of enzyme enantioselectivity, but also shed light on the origin of homochirality.

## 1. Introduction

Chiral homogeneity, especially the origin of L-dominant amino acids in the biological world, has intrigued scientists for many years, though there has been no general consensus as yet to the origins of homochirality. There have been many reports on natural-occurring chiral breaking due to asymmetric decomposition with hydrolysis or circular polarized light [1]. However, the half-life of biological amino acid racemization in aqueous solution is 10^3^ years for phenylalanine or 10^5^ years for glutamic acid at 298 K [2]. On the other hand, the duration needed for the emergence of life is more than seven million years [3]. Even if one-handed amino acids and sugars had just happened to appear on earth with only a slight enantiomeric excess before the creation of life, any slight excess would have been quickly extinguished by racemization in water [4]. Life might have not existed if enantiomers had been heterogeneous at an earlier stage of the creation of life. Even now, racemization pressure is still overwhelmingly strong in the natural environment, especially in aqueous solutions. Amino acids (also sugars) are stereochemically too labile to keep their enantiomeric type homochiral in the natural environment. It is thought that life, which requires homochirality, had to create a new elaborate mechanism to generate it in an early stage. It remains unknown, but the present biological world is in a homochirally-stable state. For example, amino acids, which can be regarded as components of all polypeptides, must be homogeneous to assemble a regularly structured polypeptide. If a polypeptide is mixed with L- and D- amino acids, it is no longer possible to create an ultra-precise structure because the racemic mixture of enantiomers cannot elongate the polypeptide chain [5]. In this context, life itself can be regarded as an ultimately homoorganized structure made up of homochiral biomolecules. Life’s first strategy is to homogenize racemic amino acids or sugars to utilize them as the components of protein or nucleic acid. Perhaps some sophisticated contrivance is required to produce homochiral amino acid molecules enough to synthesize polypeptides. At least a systematic mechanism with automatically developing chiral homogeneity [6] is thought to exist, and additionally, it is expected that there is a very complicated mechanism that selects and delivers the right enantiomeric type to the right place while at the same time excluding the opposite enantiomer in order to synthesize regularly structured macromolecules. By the time catalytically active early polypeptides, *i.e.*, primitive enzymes, were present, the switch to the exclusive use of L-amino acids might already have been made. If early polypeptides were thus synthesized by spontaneous abiotic processes in a primitive racemic environment, the mechanism of homochirality may have already been incorporated into them, and their descendants may be traced to present enzymes. Such a mechanism is involved in enzyme enantioselectivity in extant life. It must be absolutely stable because unstable enantioselectivity would mean the end of life. We can thus say life does not exist without homochirality, *i.e.*, no homochirality—no life [7]. This principle is so fundamental that it is inseparably associated with the origins of life. The substrate specificity of many enzymes for L-amino acids and their catalytic mechanisms are studied with the corresponding substrates, while the mechanism of enantioselectivity regarding physiological active D-amino acids is only poorly understood. A deeper understanding of the enzyme enantioselectivity is important to elucidate the origin of homochirality.

A stable supply of homochiral molecules, which is necessary for synthesizing biological macromolecules with highly organized stereostructures such as proteins or polysaccharides, is achieved by enzyme enantioselectivity that functions to select the right enantiomer; and therefore the stability of this enantioselectivity is essential for sustaining vital activity [8]. Enantioselectivity is generally understood to be stable. We have so far studied the enantioselectivity of tryptophanase, with the aim of acquiring a better understanding of the enantioselectivity. Tryptophanase is an enzyme that degrades L-tryptophan into indole, pyruvate and ammonia, or synthesizes L-tryptophan from L-serine and indole. It is also known as an enzyme that has a very wide substrate specificity for L-tryptophan derivatives and various β-substituted L-amino acids, but has an extremely tight enantioselectivity [9]. Because of this absolute enantioselectivity, tryptophanase has no activity on D-tryptophan and D-serine at all. However, previous studies have shown that tryptophanase becomes active towards the D-enantiomers in highly concentrated diammoniumhydrogen phosphate ((NH_4_)_ 2_HPO_4_), DAP for short) solution [10,11]. Tryptophanase enantioselectivity is very flexible in the presence of DAP, contrary to conventional knowledge about enantioselectivity. D-tryptophan or D-serine, respectively, was degraded or synthesized through β-elimination or β-replacement reactions after D-tryptophan or D-serine formed an aldimine bond with pyridoxal 5'-phosphate [12]. The formation of the external aldimine bond between the D-enantiomers and pyridoxal 5'-phosphate was the first step for D-enantiomers to function as active substrates. However, even if C^α^-NH_2_ succeeded in the formation of the external aldimine bond with pyridoxal 5'-phosphate, subsequent reactions did not always proceed automatically. Several inhibitors with amino groups, for example, L-phenylalanine or L-methionine, can also form the external aldimine bond with pyridoxal 5'-phosphate, but they can never become substrates for tryptophanase [13]. This indicates that the forming of the aldimine bond is essential to react with D-enantiomers, it is not enough for the flexible tryptophanase enantioselectivity to arise in the presence of DAP. Perhaps other factors such as conformational change are required as well. Previous reports showed that tryptophanase underwent a small reversible conformational change in the presence of DAP [14]. We think this small conformational change possibly modifies the enantioselectivity of tryptophanase so that D-enantiomers can be activated *via *the external aldimine bond formation. Inhibitors, which structurally resemble their enzymes’ substrates but either do not react or react only very slowly compared to the substrate, are commonly used to probe the conformational nature of a substrate-binding site to elucidate the enzymes’ catalytic mechanisms. Therefore, when a tryptophan-analogous inhibitor is used for kinetic analysis, it is powerful enough to understand what happens at the active site of the tryptophanase with a conformational change [15]. This report studies the reason why the flexible enantioselectivity emerges in the presence of DAP.

## 2. Results and Discussion

### 2.1. Effect of DAP and D-Tryptophan on L-Tryptophan Degradation Reaction

**Figure 1:**
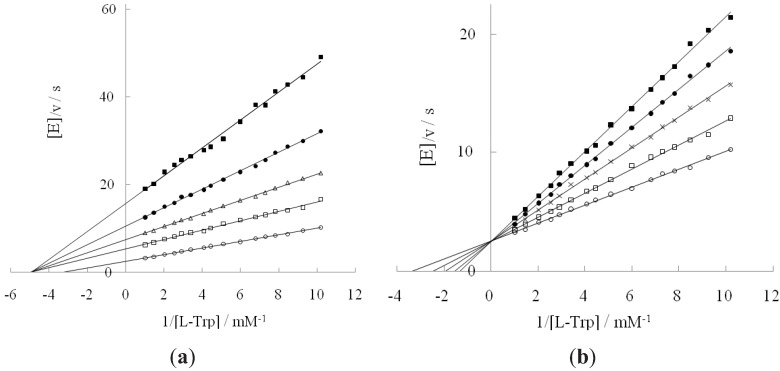
Inhibition of diammoniumhydrogen phosphate (DAP) (**a**) or D-tryptophan (**b**) against L-tryptophan degradation reaction. (**a**): DAP concentrations: 0 M (○); 0.6 M (□); 1.2 M (∆); 1.9 M (●); 3.1M (■); (**b**): D-tryptophan concentrations: 0 mM (○); 9.8 mM (□); 14.7 mM (×); 19.6 mM (●); 24.5 mM (■).

It is reported that tryptophanase reacts with D-tryptophan in the presence of DAP [16]. We first investigated how DAP interacted with the enzyme in L-tryptophan degradation. In Figure 1a, four plots (□, ∆,●, ■) were separated from a plot (○) because the Km value for L-tryptophan shifted from 0.3 to 0.2 mM in the presence of DAP, along with reduced values of k_cat_ (18–45% ) and catalytic efficiency (26–68%) compared to the values in the absence of DAP (Table 1). When suppressing the L-tryptophan degradation reaction as a noncompetitive inhibitor, DAP had a higher degree of affinity for L-tryptophan. The inhibition constant of DAP was 640 mM. This value was considerably higher than the values of the other inhibitors used in this study. Mild noncompetitive inhibition behavior of DAP will cause any small conformational change to deviate the activity onto D-tryptophan. Although we investigated other salts including ammonium and phosphate ions elsewhere [14], they could not activate D-tryptophan by just effecting some change in conformation. Both ammonium and phosphate ions are necessary to provoke the activity of tryptophanase on D-tryptophan.

**Table 1:** Kinetic parameters for L-tryptophan degradation in the presence of DAP and Ki, Ki' values of D-tryptophan.

DAP concn./M	k _cat_/s	Km/mM	k _cat_/Km/M∙s	Inhibition const./mM		Inhibition type
				Ki	Ki'	
0	0.40	0.3	1.3×10^3^	21	-	competitive
0.6	0.18	0.2	0.9×10^3^	25	128	mixed
1.2	0.15	0.2	0.8×10^3^	30	90	mixed
1.9	0.11	0.2	0.6×10^3^	38	56	mixed
3.1	0.07	0.2	0.4×10^3^	49	49	noncompetitive

Since enzymes are frequently referred to as ideal examples of enantioselectivity, we usually think that they should have no interaction with D-enantiomers. However, many enzymes do not show this behavior. In fact, they can bind both enantiomeric forms of a substrate [17]. The consequences of such binding behavior vary depending on the enzyme. While the catalytic reaction proceeds with both forms as is the case for the amino acid and hydroxyl acid racemases, it is restricted to one enantiomer in other enzymes. Especially in the latter case, another enantiomer is inert or functions as an inhibitor even though it is bound to the active site. Most amino acid-catalyzing enzymes show this reactivity behavior. The D-enantiomer is inactive or inhibits enzyme, whereas the L-enantiomer is active. The same is true in the case of tryptophanase. Figure 1b shows the inhibition action of D-tryptophan on L-tryptophan degradation in the absence of DAP. D-tryptophan functioned as a competitive inhibitor when it was bound to tryptophanase. The Ki value for D-tryptophan was 21 mM.

### 2.2. Inhibition Behavior of D-Tryptophan in the Presence of DAP

**Figure 2:**
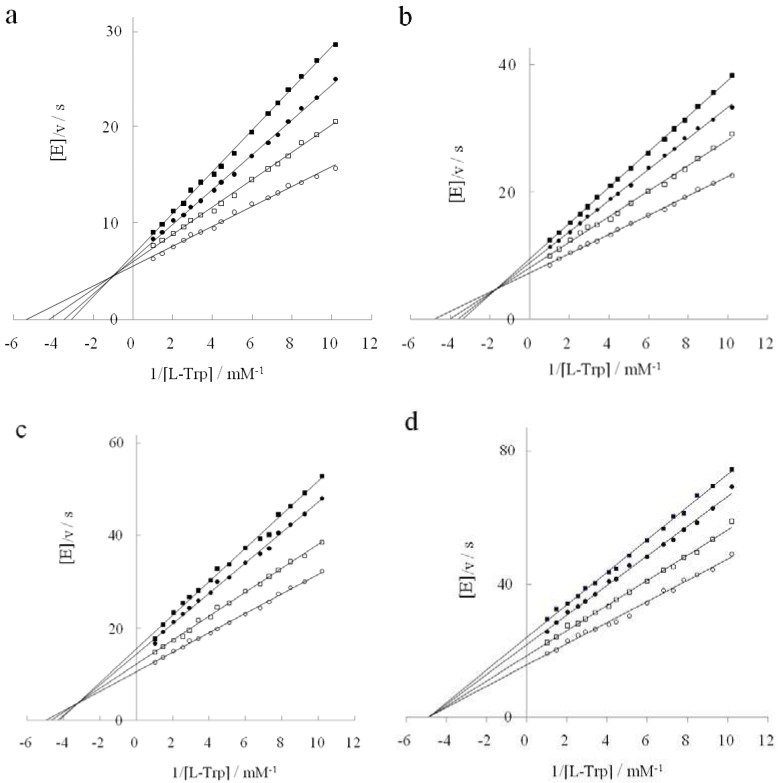
Inhibition reaction of D-tryptophan shifting with increasing DAP concentrations. DAP concentration: (**a**): 0.6 M, (**b**): 1.2 M, (**c**): 1.9 M, (**d**): 3.1 M. D-tryptophan concentration: 0 mM (○); 9.8 mM (□); 19.6 mM (●); 24.5 mM (■).

**Figure 3:**
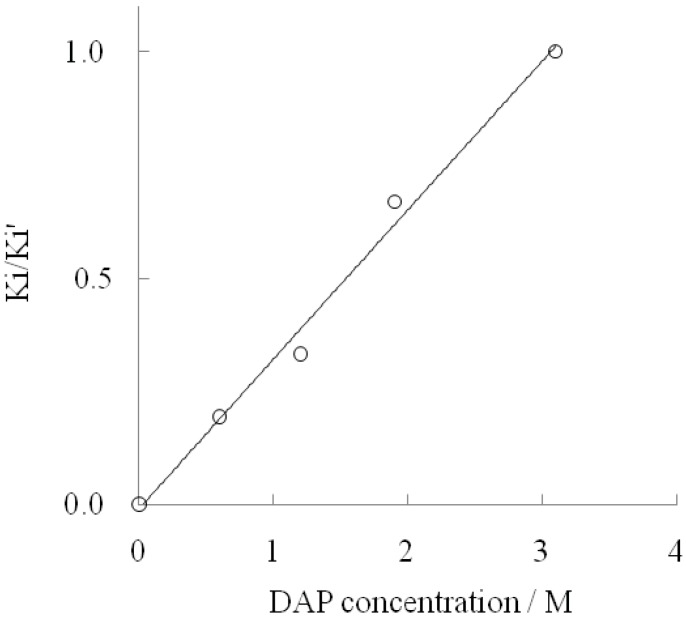
Increasing Ki/Ki' with DAP concentrations.

While DAP itself exerts a noncompetitive inhibitory action on tryptophanase in the L-tryptophan degradation reaction, D-tryptophan serves as a competitive inhibitor in the absence of DAP. Thus we investigated whether the inhibitory behavior of D-tryptophan changed in the presence of DAP. Figure 2 shows how the inhibitory behavior changed with DAP concentrations. Kinetic parameters for D-tryptophan are summarized in Table 1. Figures 2a–c show a mixed type inhibition with a fixed point shifting left-downward into the second quadrant with increasing DAP concentrations. Since the x and y coordinates of the fixed point were (−[D − Trp]/Ki')/(Km[D − Trp]/Ki) and (1/Ki − 1/Ki')[D − Trp]/(Vm[D − Trp]/Ki), Ki and Ki' values were determined from these coordinates. While Ki increased with increasing DAP concentrations, Ki′ decreased. The inhibitor (D-Trp) could bind to either the enzyme E or the enzyme-substrate complex ES with different inhibition constants. Although D-tryptophan potently competed with L-tryptophan at the tryptophanase’s active site in the absence of DAP, it was weakened gradually with increasing DAP concentrations. Figure 2d shows the plots of a noncompetitive inhibition type seen when the DAP concentration was at 3.1 M. In Figure 3, Ki/Ki' increases from 0 to 1.0 with increasing DAP concentrations. The inhibition by D-tryptophan of the L-tryptophan degradation shifted from competitive to noncompetitive *via* mixed type inhibition. As opposed to the mixed type inhibition where the binding of a substrate or inhibitor affects an enzyme’s binding affinity for the other molecule, noncompetitive inhibition does not affect the binding of the substrate, although the binding of the inhibitor to the enzyme is reduced. The noncompetitive inhibition type is generally considered to result from an allosteric effect where the inhibitor binds to a different site on the enzyme [18]. We showed that DAP loosened the binding affinity of D-tryptophan at the active site of tryptophanase but reinforced it at another site different from the active site. The previous report indicated that tryptophanase activity towards D-tryptophan was at its maximum at a concentration of 3.1 M DAP [19]. When the binding of D-tryptophan with the enzyme was the weakest at the active site and the strongest at the alternative site, the activity of D-tryptophan was the highest. In this context, the increase of tryptophanase activity on D-tryptophan seems to be linked to the binding of D-tryptophan to the alternative site. If this site coincides with an allosteric site, it is very interesting. However, the allosteric site is not catalytic by definition, and so we propose the following hypothesis: Perhaps DAP exerts a noncompetitive inhibitory influence over the active site of tryptophanase and thereby induces a mild and small conformational change, which is also optimal for both substrate-binding and -catalysis of D-tryptophan.

### 2.3. Inhibition Type of Two Tryptophan Analogues

**Figure 4:**
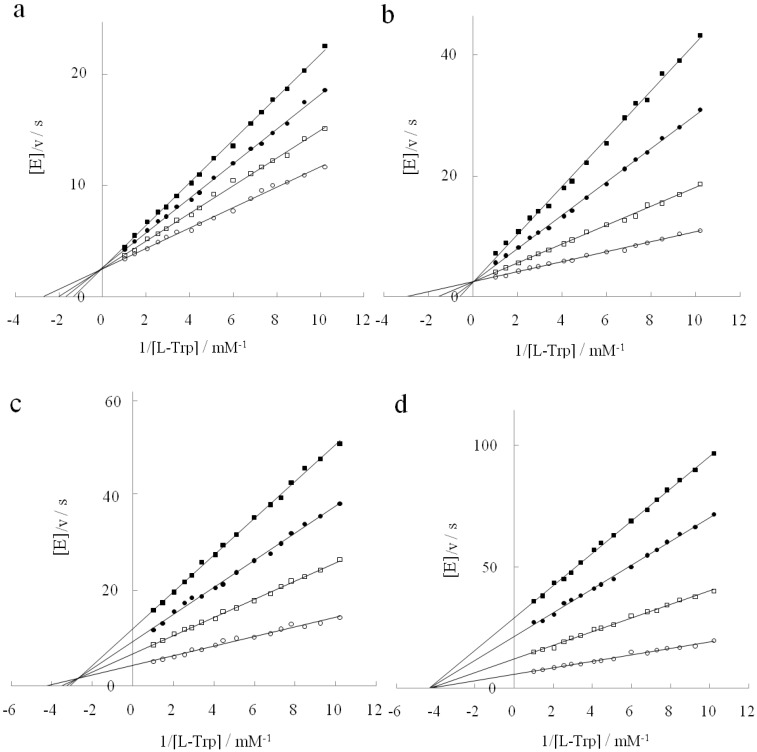
Inhibitory type of potassium pyruvate in the absence of DAP (**a**) and indole pyruvate in the presence of DAP (**b-d**). (**a**): Potassium pyruvate concentration: 0 mM (○); 1 mM (□); 1.9 mM (●); 2.9 mM (■).(**b–d**): Indole pyruvate concentration: 0 mM (○); 0.2 mM (□); 0.4 mM (●); 0.6 mM (■). DAP concentration: (b):0 M, (c): 0.6 M, (d) 1.2 M.

The inhibition pattern of D-tryptophan for L-tryptophan degradation changed with DAP concentrations. Potassium pyruvate and indole pyruvate, which are partially analogous to tryptophan, were used to study this process. Since pyruvate is the last product released, it was expected that it would bind competitively with L-tryptophan. In fact, potassium pyruvate competitively inhibited L-tryptophan degradation in the absence of DAP, as shown in Figure 4a. The same result was obtained in the presence of DAP (data not shown). On the other hand, indole pyruvate is a tryptophan analogue with a heterocyclic indole ring bound to the methyl group of pyruvate, so it would also be expected to bind competitively with L-tryptophan. However, this was not actually the case. Indole pyruvate showed the same inhibition behavior as D-tryptophan with increasing DAP concentrations (Figure 4b-d). Ki and Ki' values for the two inhibitors are shown in Table 2. Ki for potassium pyruvate was 3 mM, indicating that potassium pyruvate was a stronger inhibitor than D-tryptophan. Since pyruvate is an end product of L-tryptophan degradation, it is natural that pyruvate serves as a strong competitive inhibitor. On the other hand, Ki for indole pyruvate was 15-fold stronger than for pyruvate. This result indicates that indole pyruvate is more analogous to L-tryptophan than to D-tryptophan. Nevertheless, unlike potassium pyruvate, the inhibition pattern of indole pyruvate changed with DAP in the same manner as it did for D-tryptophan. Since the chemical structural difference between potassium pyruvate and indole pyruvate lies in the heterocyclic indole ring, there is no doubt that this moiety is responsible for this shifting of inhibition patterns in the presence of DAP. 

**Table 2:** Ki and Ki' for two inhibitors, potassium pyruvate and indole pyruvate.

inhibitor	substrate	DAP concn./M	Inhibition const./mM		Inhibition type
			Ki	Ki'	
potassium pyruvate	L-Trp	0	3	-	competitive
indole pyruvate	L-Trp	0	0.2	-	competitive
	L-Trp	0.6	0.2	0.3	mixed
	L-Trp	1.2	0.2	0.2	noncompetitive

L-Trp: L-tryptophan.

### 2.4. Inhibitory Action of D-Histidine with Pentagonal Heterocyclic Ring

Since the structural difference between D-tryptophan and indole pyruvate is a C^α^-bound hydrogen and amino group, there is the possibility that this group—in addition to the heterocyclic moiety of D-tryptophan—participates in the change of the inhibition behavior with DAP. D-phenylalanine, D-thyrosine and D-histidine, which are of a similar chemical structure as D-tryptophan because their side group has a hexagonal or heterocyclic aromatic group, were thus preliminarily investigated in order to study whether these amino acids inhibited the L-tryptophan degradation reaction in the absence and presence of DAP, and subsequently how they affected the D-tryptophan degradation reaction in the presence of 3.1 M DAP. The results showed that they had no effect on L-tryptophan degradation. This shows that the heterocyclic moiety of D-tryptophan is necessary for the inhibition behavior instead of a C^α^-H, -NH_2_, hexagonal or pentagonal ring.


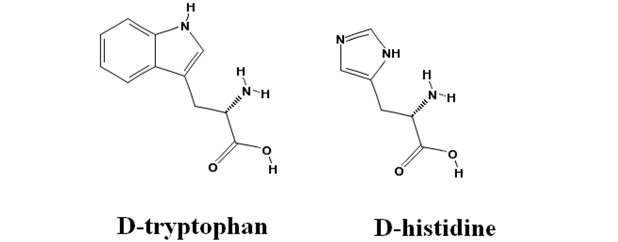


**Figure 5:**
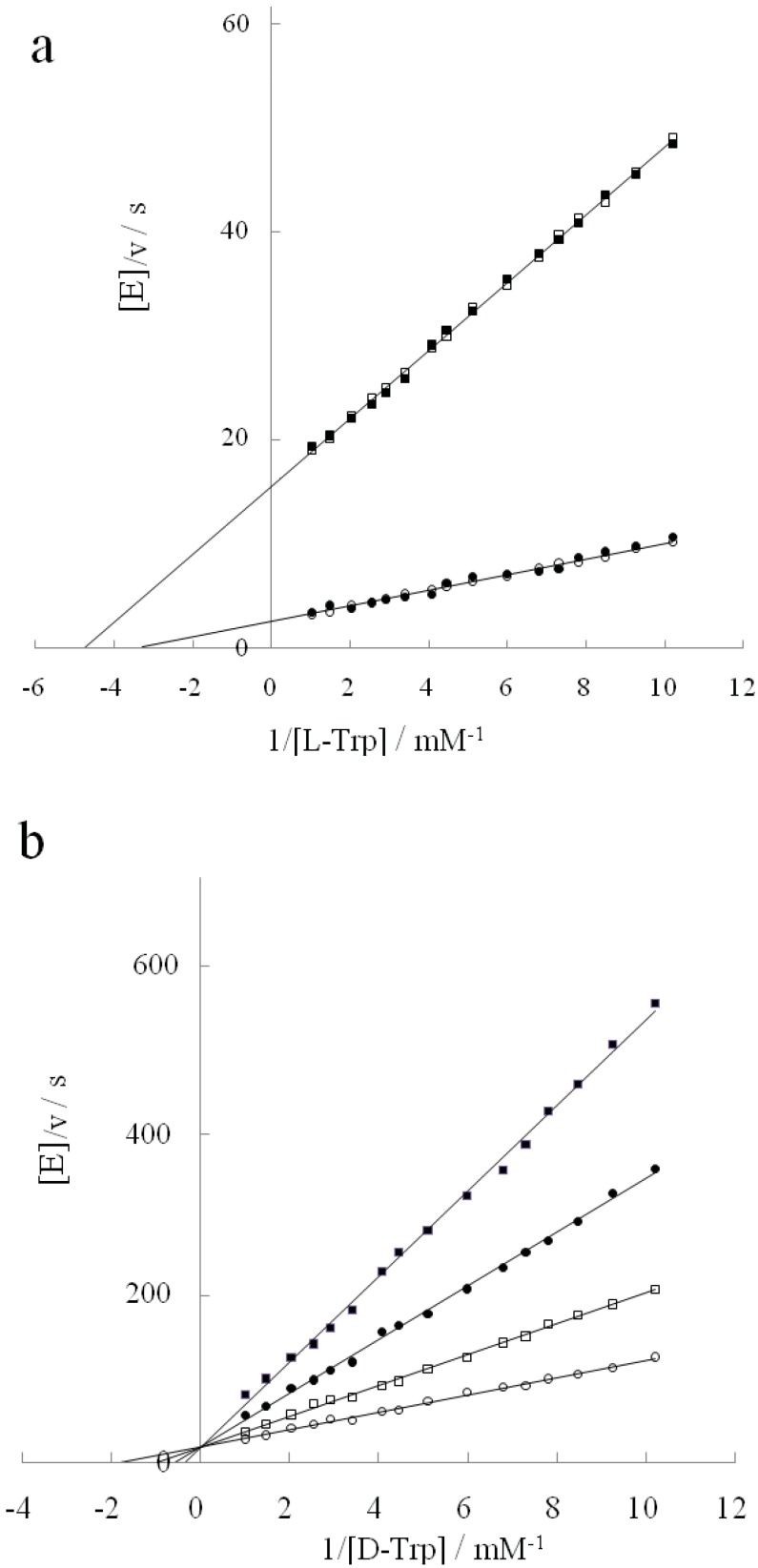
Inhibitory behavior of D-histidine. (**a**): Double reciprocal plots for L-tryptophan degradation at a DAP concentration of 0 M (○, ●) and 3.1 M (□, ■). D-histidine concentration: 0 mM (○, □); 2 mM (●, ■); (**b**): Double reciprocal plots for D-tryptophan degradation at a DAP concentration of 3.1 M. D-histidine concentration: 0 mM (○), 0.7 mM (□), 1.3 mM (●), 2 mM (■).

On the other hand, D-histidine only inhibited D-tryptophan degradation whereas both D-phenylalanine and D-tyrosine had little and no effect on it, respectively. As illustrated above, in D-histidine the indole ring found in D-tryptophan is substituted with an imidazole group. Since the NH group in a different position on the ring of histidine participates in the catalytic triad [20], one may think that D-histidine is not a very good analogue of D-tryptophan. However, we consider imidazole to be comparable in magnitude to the pyrrol ring of indole in terms of its chemical structure. Thus we examined the inhibition pattern of D-histidine. D-histidine competitively inhibited D-tryptophan degradation in the presence of 3.1 M DAP (Figure 5b), while it did not interact with L-tryptophan in the absence or presence of DAP (Figure 5a). Kcat and Km for D-tryptophan degradation were 0.07 /s, and 0.6 mM, respectively, and Ki for D-histidine was 0.4 mM. D-histidine had a different inhibition pattern from D-tryptophan. The size difference between D-histidine and D-tryptophan lies in the benzene ring. We suggest that the side group of D-tryptophan is more bulky than D-histidine due to the benzene ring, and consequently the bulkiness makes it possible to interact with the catalytic site of tryptophanase. 

### 2.5. Flexible Enantioselectivity of Tryptophanase

Tyrosine phenol-lyase is an enzyme closely related with tryptophanase. Tryptophanase and tyrosine phenol-lyase catalyze the reversible hydrolytic cleavage of L- tryptophan or L- tyrosine to indole or phenol, respectively, and ammonium pyruvate. They are very similar in sequence and structure. Although they are known to bind various amino acids, they show strict specificity for their respective physiological substrates. When cleavage of the side chain is chemically impossible, the bound amino acid acts as a reversible competitive inhibitor. The chemical transformation in the active site ends with the generation of the corresponding quinonoid intermediate in this case [21]. If the same thing happens to D-tryptophan, the quinonoid intermediate will become a dead end. D-tryptophan is nothing but a competitive inhibitor in the absence of DAP, even if D-tryptophan binding in the active site can be assumed to induce the conformational changes that depend on the structure of the side chain and affect the relative orientation of the C^α^-H of the external aldimine and the ε-amino group of the acceptor lysine residue. When DAP is added, DAP provides enough conformational change to noncompetitively inhibit tryptophanase. The additional conformation change induces a change in their relative orientation that is certainly capable of promoting the deprotonation of C^α^-H. As a result, D-tryptophan is degraded in the same pathway as L-tryptophan.

A superimposition of active-site residues taken from the spatial structures of tyrosine phenol-lyase in complex with 3-(4'-hydroxyphenyl)propionic acid and tryptophanase holoenzyme was presented on the basis of crystallographic studies [22,23]. According to this scheme, the C^α^-H of D-tryptophan occupies the symmetrical mirror opposite position to the one of L-tryptophan even though the other three residues bound to the α-carbon are superimposing one another. Since a steric configuration of D-tryptophan cannot superimpose one of L-tryptophan, this makes it difficult to explain why D-tryptophan becomes active. In the present study, D-histidine inhibited only D-tryptophan degradation but had no effect on L-tryptophan degradation. This raises the possibility that the active site of D-tryptophan is partially independent of that of L-tryptophan, also making us imagine something like a pocket into which D-tryptophan slides in the immediate vicinity of L-tryptophan bound to PLP in the active site. Studies on the crystal structure of L- or D-serine dehydratase help to interpret our results. In the case of L-serine dehydratase, Lys41 abstracts the proton from the C^α ^of L-serine in a concerted fashion, whereas in D-serine dehydratase Thr168 is responsible for the abstraction [24,25]. The role of the proton abstraction from the C^α^ of L-tryptophan is played by Lys270 in tryptophanase. If tryptophanase is the same as the serine dehydratase, the abstraction of that of D-tryptophan will be performed by any other possible amino acid with high basicity in the above-mentioned pocket. Our results support a small conformational change in the enzyme from the native L-conformation to an alternative D-conformation at high DAP concentrations. Since L-tryptophan binds to the L-conformation and adversely the D-conformation binds to the other, they are not competitive, but show either mixed or noncompetitive inhibition in the presence of DAP. For example, thyrosine phenol-lyase has been demonstrated to have two distinct conformations that bind L-alanine and D-alanine, respectively [26]. The switch from L-conformation to D-conformation is controlled by DAP, and this fact will offer interesting insights into the origin of homochirality. Today’s saline environment in aqueous solution may give an optimal condition for the exclusive use of the L-conformation, and consequently the L-amino acid may become dominant in the contemporary biological world. 

The reaction process of α-proton abstraction and indole dissociation from L-tryptophan is quite complicated because of the involvement of the different acid-base catalyses of side chains of amino acids in tryptophanase. We indicated that a benzene ring of D-tryptophan in addition to DAP was important for the flexible enantioselectivity of tryptophanase. D-tryptophan also goes through the complex process of acid-base catalysis to be degraded into indole and ammonium pyruvate *via* several intermediates. It will be significant to identify their intermediates in the presence of DAP in order to understand this reaction mechanism. These intermediates should be detected and characterized by spectroscopic analysis in our future study. 

## 3. Experimental Section

### 3.1. Materials and Reagents

L-tryptophan, D-tryptophan and D-histidine were purchased from Peptide Institute Inc. (Osaka, Japan), DAP, potassium pyruvate, indole and indole pyruvate from Wako Pure Chemical Industries (Osaka, Japan), pyridoxal 5′-phosphate from Nakalai Tesque Inc. (Kyoto, Japan). Apotryptophanase from *E. coli* (MW: 220000) was purchased from Sigma-Aldrich Co. (St. Louis, MO, USA), which is easily soluble in aqueous solution. One mg of apotryptophanase released 0.11 μmole of indole from L-tryptophan in a minute at 37 °C in 100 mM potassium phosphate buffer of pH 8.3 in the presence of 0.2 mM of pyridoxal 5′-phosphate. DAP was dissolved completely in 80–85 °C hot water and slowly cooled to room temperature until crystallization finished completely. Each salt solution was diluted to the required saturation concentration with 100 mM potassium phosphate buffer (pH 8.3) prior to the experiment. Unless otherwise noted, all chemicals were of a high reagent grade, purchased from Wako Pure Chemical Industries. All glassware was washed by soaking it for more than three days in a detergent, Clean 99 CL, then thoroughly rinsing and then drying it in an oven. All aqueous solutions were prepared from deionized and ceramics-distilled water.

### 3.2. Reaction Conditions

When tryptophanase degraded L-tryptophan, all reaction mixtures included 0.2 mM of pyridoxal 5′-phosphate and 0.23 μM of tryptophanase in 100 mM potassium phosphate buffer of pH 8.3. DAP concentrations were prepared to the required concentrations of 0, 0.6, 1.2, 1.9 or 3.1 M. L-tryptophan. The reaction mixture was prepared to a concentration of 98, 108, 128, 137, 147, 167, 196, 225, 245, 294, 343, 392, 490, 680 or 980μM for kinetic analyses. Reactions were performed at 37 °C for 30 min in a Dry Thermo Unit DTU-1B (Taitec, Tokyo, Japan). Although D-tryptophan in addition to L-tryptophan was also degraded into indole in the presence of DAP, an indole production amount from D-tryptophan was neglected at this reaction temperature and time because the catalytic efficiency for D-tryptophan was 0.3% of that for L-tryptophan. On the other hand, the reaction condition of D-tryptophan degradation differed in several ways from that of L-tryptophan degradation. Each concentration of pyridoxal 5′-phosphate and tryptophanase was 1.2 mM and 0.92μM, and additionally reaction temperature and time was 55 °C and 2 h, respectively. All the other reaction conditions were the same as in the L-tryptophan degradation. 

The total volume of the reaction mixture was 2 mL per tube. The reaction was stopped by adding 2 mL of n-butanol, which was vigorously mixed and then immediately centrifuged at approximately 1,000 G for 10 min. After centrifugation, the reaction mixture was separated into two layers of butanol and aqueous solutions in which indole was equally distributed. The supernatant butanol layer was extracted to be mixed with an equal volume of Ehrlich reagent, then colored red at 60 °C for 30 min in Dry Thermo Unit. Since this red matter had a wavelength of a maximum absorption at λ = 570 nm, tryptophanase activity was calculated from absorbance determination there. Tryptophanase activity was assayed in triplicate at the same substrate concentration and then averaged. Initial velocity was computed based on the averaged value.

### 3.3. Kinetic Assay

Initial velocities were defined by indole that formed within 1 minute after 1 mg of tryptophanase was added. Then the reciprocal of (initial velocity/[E] (enzyme concentration)) (ordinate) versus the reciprocal of substrate concentration (abscissa) was drawn for each concentration of L- or D-tryptophan. These double-reciprocal plots were drawn based on the least squares method to calculate kinetic parameters. K_cat_was determined from the reciprocal of the intercepts of these double-reciprocal plots, and the Michaelis constant Km from substrate concentration at 1/v = 0 in the absence of inhibitor. In noncompetitive inhibition, two inhibition constants of Ki (=[E][I]/[EI], E: enzyme; I: inhibitor; EI: enzyme-inhibitor complex) and Ki' (=[ES][I]/[ESI], ES: enzyme-substrate complex; ESI: enzyme-substrate-inhibitor complex) were determined from the slopes and from the intercepts, respectively. In the mixed type inhibition, Ki and Ki' were determined from x and y coordinates of the fixed point in the second quadrant.

## 4. Conclusions

Tryptophanase is one of the enzymes with absolute enantioselectivity, but it reversibly changes in concentrated DAP solution. This flexible enantioselectivity is caused by a small conformational change in the presence of DAP. When D-tryptophan, pyruvate, indole pyruvate and D-histidine were used as inhibitors, their inhibition patterns were examined in terms of kinetics to see how the conformation affected the activity of tryptophanase on L-tryptophan degradation. The inhibition of D-tryptophan shifted from competitive to noncompetitive *via* mixed type inhibition with increasing DAP concentrations. This inhibition pattern was also obtained from experiments with indole pyruvate. We indicated that a heterocyclic benzene ring of D-tryptophan was responsible for the inhibition pattern on the basis of experimental results using D-histidine. Additionally, we suggested that tryptophanase had two different conformations (L-conformation and D-conformation) between which it switched depending on the saline environment in aqueous solution. Our results shed light on the origin of homochirality; that is to say they provide the possibility that today’s exclusive use of L-amino acids in the biological world might be associated with the saline environment *in vivo*. This study provides an attractive mechanism to explain the origin of homochirality.
